# Resilience in the face of emergency remote teaching: EAL pupils’ experiences during the COVID‐19 pandemic

**DOI:** 10.1002/tesj.591

**Published:** 2021-06-02

**Authors:** Nathan Thomas, Gabor Lucski, Erika McCulloch

**Affiliations:** ^1^ UCL Institute of Education; ^2^ University of Oxford; ^3^ Birkbeck, University of London

## 1 INTRODUCTION

Schools worldwide have been affected by the COVID‐19 pandemic. Teachers had to transition to online lessons. Pupils, too, were forced to adapt to distance education and the lack of social interaction that brick‐and‐mortar schools can provide. International schoolchildren who returned home had to contend with online classes in different time zones, technological issues, and growing concerns over physical and mental well‐being.

With the above driving the current study, we report on how English as an additional language (EAL) pupils enrolled in a preparatory school in Oxford, England coped with emergency remote teaching (ERT). In the United Kingdom, preparatory schools are independent primary schools for children up to 13 years old. Like others globally, these pupils had to study online beginning in late March 2020. Adapting the definition provided by Hodges, Moore, Lockee, Trust, and Bond (2020), we define ERT as a temporary shift from on‐site instruction to online education due to crisis circumstances. Theoretically, we draw on self‐determination theory and the concept of secondary control.

## 2 THEORETICAL FRAMEWORK

Self‐determination theory (SDT) is a theory of human behavior that charts motivation on a continuum (controlled—autonomous). SDT “is particularly concerned with how social‐contextual factors support or thwart people’s thriving through the satisfaction of their basic psychological needs for competence, relatedness, and autonomy” (Ryan & Deci, 2017, p. 3). *Competence* refers to one’s ability to succeed at a task; *relatedness* represents one’s feelings of connectedness with others; and *autonomy* relates to one’s need to feel in control of one’s experiences and actions. Satisfying one’s basic psychological needs typically leads to higher‐quality motivation, better learning outcomes, and positive well‐being (Ryan & Deci, 2017). Moreover, greater psychological well‐being is associated with improved adaptive decision making (Páez‐Gallego, Gallardo‐López, López‐Noguero, & Rodrigo‐Moriche, 2020). Because pupils were forced to participate in a situation (ERT) external to their primary control, it is important to explore well‐being (via basic psychological needs) in conjunction with adaptive decision making (secondary control).

Secondary control relates to adjusting internal factors (changing oneself) to adapt to one’s environment (Weisz, Rothbaum, & Blackburn, 1984). By applying concepts from SDT and secondary control to language learning, Chaffee, Noels, and McEown (2014) showed how learners demonstrated resilience (maintained motivation and positive affect) using secondary control (namely, positive reappraisal), despite studying with highly controlling teachers. Following Chaffee et al. (2014, pp. 357–360), we operationalized three subtypes of secondary control:

- *Positive reappraisal*. Attempts to construe existing realities to derive a sense of meaning or purpose from them and thereby enhance one's satisfaction with them.
- *Lowering aspirations*. Attempts to predict events and conditions accurately to control their potentially negative impact on oneself.
- *Vicariousness*. Attempts to associate or closely align oneself with other individuals, groups, or institutions to participate psychologically in their control.

Theoretically, if pupils’ basic psychological needs are satisfied, they will be better able to exercise their secondary control to cope with and progress in EAL lessons. In the current study, we use SDT’s basic psychology needs (competence, relatedness, and autonomy) and three subtypes of secondary control (positive reappraisal, lowering aspirations, and vicariousness) as a framework for understanding EAL participants’ experiences with ERT.

This study addresses the following research questions:

1. Are EAL pupils’ basic psychological needs being met with ERT? If yes, how?
2. Do EAL pupils use secondary control strategies to cope with ERT? If yes, how?

## 3 METHODOLOGY

### 3.1 Context

The study took place at a preparatory school in Oxford, England. Approximately 600 pupils study in Grades 4–8, of whom roughly 6% are required to participate in 30–90 minutes of one‐to‐one EAL instruction weekly. Normally, this instruction occurs during non‐core lessons such as Art, Games, or PE.

Due to precautions stemming from COVID‐19, all instruction moved online in March 2020. The transition took place during a 3‐week break, which provided time for the information technology infrastructure to be established. Teachers used Zoom for live lessons and Firefly to set tasks for pupils. Most international pupils had returned to their home countries.

### 3.2 Participants

EAL pupils who had attended 5+ ERT sessions were invited to participate. Consent was obtained from the school’s senior leadership, safeguarding, and compliance teams; participating pupils; and the pupils’ parents. Participant information is presented in Table 1. CEFR scores are based on internal assessments conducted by the school’s EAL department.

**TABLE 1:** Participant information

Participant	Grade	Age	Sex	Nationality	CEFR
P1	4	9	F	Chinese	B1
P2	4	9	M	Nepalese	B1
P3	7	12	M	Chinese	B1
P4	7	12	F	Kazakh	B2
P5	8	13	M	Kazakh	B2
P6	8	13	M	Russian	B2
P7	8	14	M	Danish	C1

### 3.3 Data collection and analysis

Teacher‐researchers conducted interviews with their own students via Zoom (ranging from 22–32 minutes). Interviews were conducted in English, because the teacher‐researchers worked extensively with their pupils previously, understood their pupils’ level, and were able to accommodate accordingly. There was also a level of rapport not usually present when outside researchers conduct interviews in schools. First, pupils were asked about their general perceptions of EAL lessons. Then, pupils were asked specifically about ERT. Interviews were recorded, transcribed, and anonymized by the second and third authors.

The data were analyzed using a mix of deductive and inductive coding. The deductive codes align with SDT’s basic psychological needs and the three subtypes of secondary control presented above. Inductive codes were used for additional data later summarized as ancillary information. The first and second authors coded the data separately; disagreements were discussed; and final coding decisions were checked by the third author.

## 4 FINDINGS

### 4.1 Basic psychological needs

Participants reflected positively on their remote EAL lessons, finding them beneficial. As indicated by the pupils’ comments, their basic psychological needs appeared to be relatively satisfied. For example, P6 described the lessons as “helpful … the same as at school,” to which P7 added, “they definitely improved my English; that’s just a fact.” These comments demonstrate how EAL lessons helped to satisfy the basic psychological need of competence, although ERT did impair some pupils’ perceived competence when technological issues occurred. For instance, P2 often experienced login issues (or “system issues” [P7]) and internet glitches that interrupted his lessons, as did P3 and P5, who, along with P6, expressed feeling more distracted at home. However, these feelings were not shared by all. P4, who did not indicate experiencing technological setbacks, explained that “it is so much easier to concentrate when you are relaxed [at home].” This suggests that perceived competence could be affected by the nature of one’s technological infrastructure and home environment.

Overall, all pupils felt some degree of control over lesson content and comfortable asking questions. As P6 noted, “I had some problems …, and I asked you to help me. … I was able to choose what we could do in our lessons.” Surprisingly, challenges regarding when lessons could occur due to time differences were reported by only two pupils (P2, P3).

Potentially troubling, however, is the low occurrence of data pertaining to relatedness, especially since most pupils were in different countries. Only P3 and P7 reported communicating with other EAL pupils online. P2, the most extreme case, mentioned not communicating with *any* pupils. Instead of playing with friends during breaks at school, he said “I’m sitting at home doing nothing.” He added that it would be nice “just to talk to them,” seemingly disconnected from others.

### 4.2 Secondary control

In terms of how pupils initiated secondary control, only P6 and P7 expected ERT to be worse than in‐person lessons (lowering aspirations), although they were actually “about the same” (P6) and P7 “really enjoyed it.” The other pupils expected ERT to be the same (P3, P4), different but not necessarily worse (P2, P5), or thought nothing of it (P1).

There were no comments coded as clearly representative of vicariousness during ERT. This, coupled with only two mentions of lowering aspirations above, supports Chaffee et al.’s (2014) finding that these forms of secondary control may not function well as predictors of optimal language learning under imposed conditions. However, also aligning with Chaffee et al., positive reappraisal was strongly represented in the data.

Pupils positively reappraised scheduling issues in light of not having to miss “fun” (non‐core) classes. P3 and P6 were happy to schedule EAL lessons in the morning; P4 expressed that “it is better for me if I don't skip any lessons, because I find them all fun”; and P7’s lessons “fit in perfectly” with his school day. P4 also noted that

I get to be at home and be at a school, which provides really good education. … I always wished that, like, there was a [school name] in Kazakhstan. And I guess, like, this was another way of the school being in Kazakhstan.

P4’s comment links with Mittelmeier, Rienties, Gunter, and Raghuram’s (2020) discussion of internationalization at a distance programs that enable learners to “remain ‘at home’ while using technology to study with an institution or program that is simultaneously located ‘abroad’” (p. 1). However, P4 later admitted that she “wouldn't be happy if it was just online school for the rest of [her] life.”

P5 also positively reappraised ERT, despite having “better access to the board and all the equipment” at school and preferring in‐person lessons. He stated that “it’s just generally good that we still get to learn,” mentioning several times that exams were approaching; the usefulness of online lessons was “not necessarily about the content, but just that they actually exist; … they don’t go to waste.”

## 5 DISCUSSION AND CONCLUSION

This research brief discussed how EAL pupils coped with ERT. Data demonstrated how pupils’ basic psychological needs were satisfied relatively well. Supportive relationships with teachers enabled pupils to benefit from EAL (competence). Participants seemed to internalize their control over lesson aims and content (autonomy) and maintained generally positive well‐being, despite indicating comparatively low levels of closeness with peers (relatedness). We surmise that need‐supporting school and home environments provided relatedness to some extent, although low peer‐supportive behavior still needs to be addressed.

It was apparent that when ERT was implemented, strong psychological foundations (satisfied basic psychological needs) enabled pupils to avoid anticipating negative outcomes (lowering aspirations) or associating with others to compensate psychologically (vicariousness). Adaptive decision making (secondary control) was evidenced in pupils’ numerous attempts to derive a sense of purpose and satisfaction from extenuating circumstances (positive reappraisal).

Although this study is limited due to its small sample and single, self‐report measure, it extends decades of research on the importance of fulfilling basic psychological needs to afford fully functioning, positive, and adaptive human behavior. For teachers in all contexts, it is important to consider student well‐being and affective factors alongside academic performance. Since online education is likely to become more generally accepted after the pandemic, it is imperative to remember issues such as access to reliable technology and sustained interpersonal relationships. As our study has shown, even one‐to‐one instruction, which is often highly sought after, is limited in the sense that it removes pupils from their peer groups, sacrificing an interpersonal need for an academic one. Ideally, online education would include time for personal attention from teachers and group sessions that focus on developing peer‐supportive behavior. We hope to see future research explore such topics.

## 6 THE AUTHORS

Nathan Thomas is a postgraduate researcher at the UCL Institute of Education and a course tutor in applied linguistics at the University of Oxford. He is mainly interested in language learning strategies and self‐/other‐regulation. He has published in leading journals such as *Applied Linguistics*, *ELT Journal*, *Language Teaching*, System, and *TESOL Quarterly*.

Gabor Lucski holds a master's degree in English language teaching from the University of Oxford and has taught in Hungary, Spain, Greece, and the United Kingdom. He is currently teaching EAL at an independent preparatory school in Oxford, England. His main research interests include bilingualism, materials evaluation, and task‐based learning.

Erika McCulloch obtained a master’s degree in applied linguistics from Birkbeck, University of London. She has a wealth of teaching experience, including teaching adults in language schools in London and Oxford. She is currently working as Head of EAL at an independent pre‐preparatory and preparatory school in Oxford, England.
